# Optimization and standardization of the culturomics technique for human microbiome exploration

**DOI:** 10.1038/s41598-020-66738-8

**Published:** 2020-06-15

**Authors:** Ami Diakite, Grégory Dubourg, Niokhor Dione, Pamela Afouda, Sara Bellali, Issa Isaac Ngom, Camille Valles, Mamadou lamine Tall, Jean-Christophe Lagier, Didier Raoult

**Affiliations:** 1 Aix Marseille Univ, IRD, AP-HM, MEPHI, Marseille, France; 2 IHU Méditerranée Infection, Marseille, France

**Keywords:** Microbiology, Medical research

## Abstract

Culturomics is a high-throughput culture approach that has dramatically contributed to the recent renewal of culture. While metagenomics enabled substantial advances in exploring the microbiota, culturomics significantly expanded our knowledge regarding the bacterial gut repertoire through the discovery and the description of hundreds of new taxa. While this approach relies on the variation of culture conditions and media, we have tested so far more than 300 conditions since the beginning of culturomics studies. In this context, we aimed herein to identify the most profitable conditions for optimizing culturomics approach. For this purpose, we have analysed a set of 58 culturomics conditions that were previously applied to 8 faecal specimens, enabling the isolation of 497 bacterial species. As a result, we were able to reduce the number of conditions used to isolate these 497 of more than a half (i.e. to 25 culture conditions). We have also established a list of the 16 conditions that allowed to capture 98% of the total number of species previously isolated. These data constitute a methodological starting point for culture-based microbiota studies by improving the culturomics workflow without any loss of captured bacterial diversity.

## Introduction

The exploration of the human gut microbiota and its interaction with human health represents a major current challenge. While high-throughput sequencing approaches enabled substantial advances for microbiota studies, their inability to identify the unknown content of microbial communities along with the need of biological material for proof of concepts experiments have permitted the rebirth of culture approaches. Of these, the culturomics, which is a high scale culture technique was developed to study the human microbiota^1^. This approach enabled to drastically expand our knowledge regarding the repertoire of human microbes through the discovery of new taxa and the identification of rare and tedious bacteria^2^. The number of prokaryotic species isolated at least once in humans as pathogens or commensals increased by 28% from 2015 to 2018, with an estimated 66.2% participation of Culturomics in the updating of this repertory^3^. By filling a part of the metagenomics dark matter through a reference catalogue of the genomes of cultivated human intestinal bacteria^4,5^ culturomics has challenged the dogma stating that most of the human microbiota is unculturable^6^. Browne *et al*. recently comforted this theory using targeted phenotypic culture and metagenomic sequencing^7^. In addition to being complementary to metagenomics in human microbiota studies, it also improves the identification of key bacteria associated with diseases, in which several species isolated by culturomics have been reported to play a role diseases and are therefore of potential therapeutic interest^6,8–11^. However, culturomics based approach remains long and tedious and requires a substantial hands-on-time. In particular, the multiplication of culture conditions renders the method labour-intensive as it requires to test an exponential number of colonies. In order to overcome the constraints of culture-dependant approaches, several efforts have been made to reduce the laboratory workload. Of these, the design of culture conditions with an optimal combination of factors promoting bacterial growth combined with an experimental approach of colony picking has been reported^12^. Alternatively, high throughput sequencing of phylogenetic markers and genome sequencing following an optimized culture-dependant approach enabled to capture a maximal diversity from gut specimen including the isolation of new taxa^13^.

We have previously drastically reduced the number of culture conditions from 212 to 18, but there is still a need to rationalize culturomics studies^6,14^. In particular, as various bacteria are currently identified as biomarkers in disorders, targeted cultures enable to provide biological material for *in vitro* experiments and ultimately bacteriotherapy. We have recently conducted a culturomics study by using 58 culture conditions, of which 40 were new. and have isolated 494 bacterial species of which 19 were new taxa^15^. The purpose of this work was to analyse the performances of the 58 conditions used from this work for optimizing the process of culturomics-based strategy.

## Results

### Evaluation of profitability and Optimisation of 58 culture conditions

**Figure 1:**
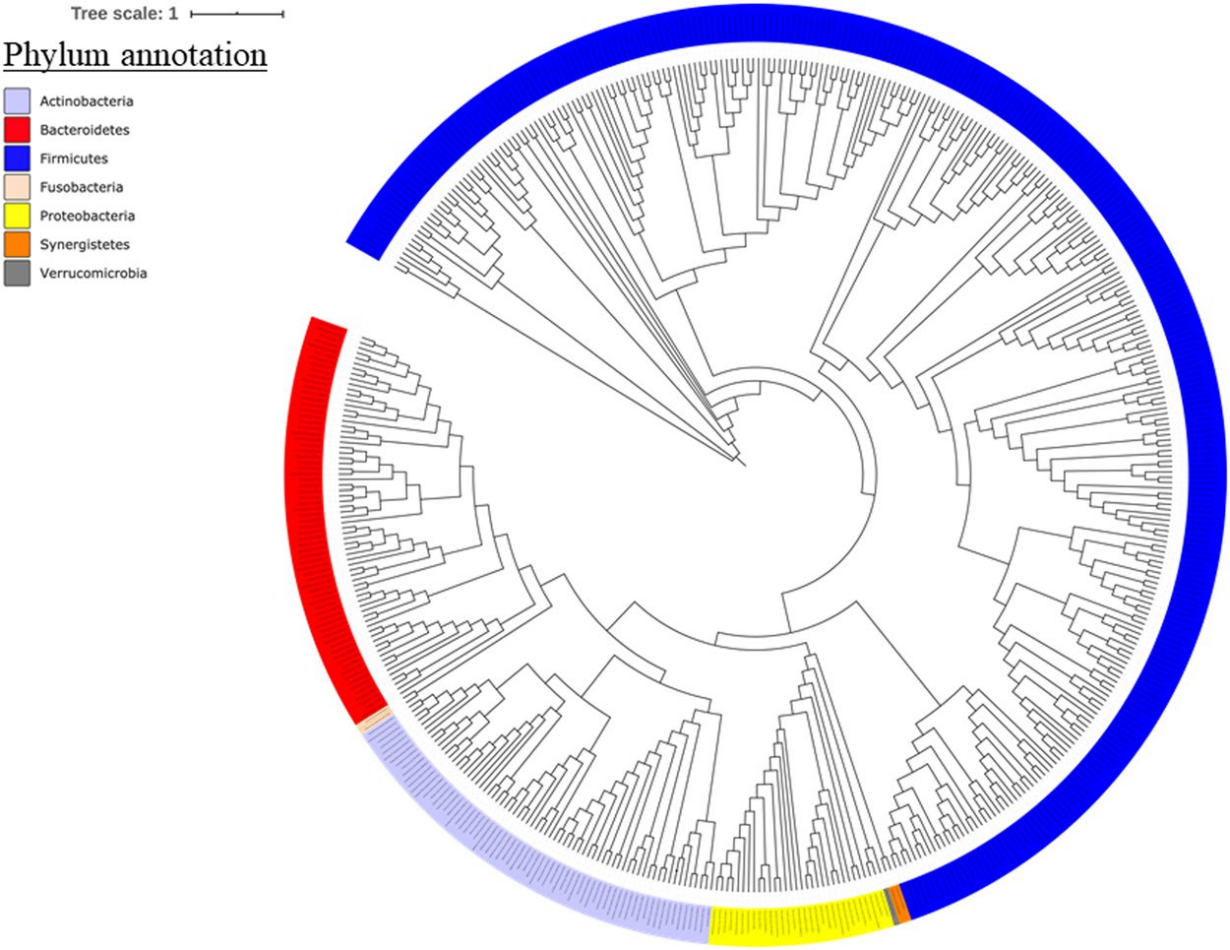
Phylogenetic tree based on the available 16S rRNA gene sequences of 480 bacterial species of this study. This image was generated thanks to the itol platform (https://itol.embl.de/).

A total of 58 culture conditions (18 aerobic and 40 anaerobic, Supplementary Table S1) were analysed and 497 species were isolated from accounting for 7 phyla (Fig. 1). These culture conditions were initially ranked according to their profitability in terms of number of species. We first established a ranking of the 15 most profitable conditions in terms of bacterial richness (i.e., number of isolated species). The blood culture bottle with rumen fluid and sheep blood in anaerobic condition at 37 °C (**HRS Ana 37 °C**) (n = 306 species) was the most profitable condition, followed by the condition R-medium with lamb serum with rumen fluid and sheep blood in anaerobic condition at 37 °C (**R-medium-SA- RS Ana 37 °C**) (n = 172 species); 5% sheep blood broth in anaerobic condition at 37 °C **(Cos Ana 37 °C**) (n = 167 species); blood culture bottle with 5 ml sheep blood in anaerobic condition at 37 °C (**HS Ana37 °C**) (n = 166 species); YCFA broth in anaerobic condition at 37 °C (**YCFA Ana 37 °C)** (n = 152 species); blood culture bottle with stool filtered at 0,45 µm in anaerobic condition at 37 °C (**Filtration 0,45 µm Ana 37 °C**)(n = 144 species); blood culture bottle in anaerobic condition at 37 °C (**Hemoc Ana 37 °C**) (n = 143 species); blood culture bottle with rumen fluid in anaerobic condition at 37 °C (**HR Ana 37 °C**) (n = 141 species); blood culture bottle after thermic shock at 80 °C during 20 min in anaerobic condition at 37 °C (**TS Ana 37 °C**) (n = 141 species); marine broth in anaerobic condition at 37 °C (**Marin Ana 37 °C**) (n = 139 species); blood culture bottle with rumen fluid and sheep blood in anaerobic condition at 37 °C after pre-treatment of stool sample with alcohol (**HRS Ana 37 °C Alcohol**) (n = 133 species); R-medium with rumen fluid and sheep blood in anaerobic condition at 37 °C (**R-medium-RS Ana 37 °C**) (n = 127 species); blood culture bottle with stool filtered at 5 µm in anaerobic condition at 37 °C (**5 µm Ana 37 °C**) (n = 126 species); Schaedler broth in anaerobic condition at 37 °C (**Schaedler Ana 37 °C**) (n = 123 species); *Christensenella* broth medium in anaerobic condition at 37 °C (***Christensenella*** **medium Ana 37 °C**) (n = 116 species) (Supplementary Fig. 1).

**Figure 2:**
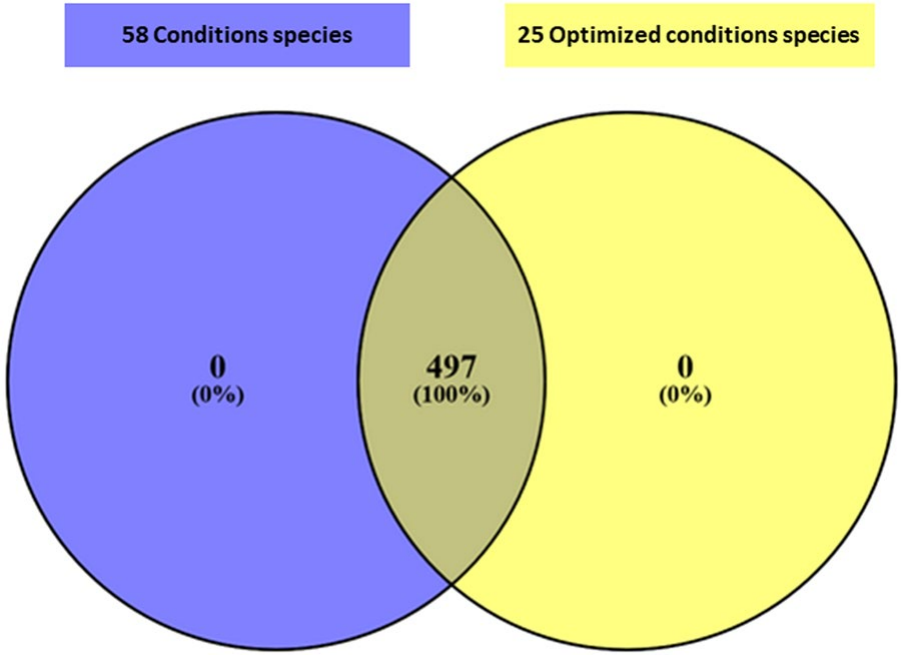
Comparison of the number of bacterial species isolated by the 58 conditions in blue VS the 25 optimized conditions in yellow.

The most useful culture conditions were determined according to their ability to grow new member previously not isolated by our best reported culture condition (i.e., **HRS Ana 37 °C**). First, we observed that 25 conditions allow to capture the entire bacterial richness from the 8 specimens (i.e., 497 species) initially found when the 58 initial conditions were applied (Fig. 2, Supplementary Figs. 1 and 2). This represents a reduction to half the number of conditions used for the same profitability in terms of number of species. A ranking of the 15 best culture conditions according to their ability to add the greatest number of previously non-isolated highlights that the **R-medium-SA- RS Ana 37 °C** is the most profitable condition (n = 64 species) following **HRS Ana 37 °C**. These two conditions are followed by the conditions **HRS Ana 37 °C Alcohol** (n = 29 species); **YCFA Ana 37 °C** (n = 21 species); **Filtration 0,45 µm Ana 37 °C** (n = 17 species); **HS Ana 37 °C** (n = 13 species); **TS Ana 37 °C** (n = 8 species); **Marin Ana 37 °C** (n = 5 species); **Filtration 0,45 µm Ae 37 °C** (n = 5 species); **5 µm Ana 37 °C** (n = 4 species); **HR Ana 37 °C Alcohol** (n = 4 species); **R-medium-RS Ana 37 °C** (n = 3 species); **CNA agar Ana 37 °C** (n = 3 species); **COS Ana 37 °C** (n = 2 species); **Schaedler Ana 37 °C** (n = 2 species) (Supplementary Fig. 2).

### Contribution of the new culture conditions used in this study

First, in order to highlight the contribution of the new culture conditions added in this study, in comparison with the 18 best conditions of culturomics previously established^6^, we grouped the culture conditions into 3 l groups. Thus, the 18 conditions, the 18 new conditions, and the 22 alcohol conditions enabled to isolate 402, 393 and 202 bacteria, respectively, accounting for a total of 497 bacteria species. The three groups share 136 (27%) species in common. Compared to the 18 standard culturomics conditions, the 18 new conditions group allows to add 77 species, 61 of which are specific, while the 22 alcohol conditions allow to add 34 species, 18 of which are specific. Overall, the 40 new conditions (18 new conditions and 22 alcohol conditions) used for the first time in this study increased the number of cultivated species by 19% compared to the 18 conditions (Supplementary Fig. 3(a)).

**Figure 3:**
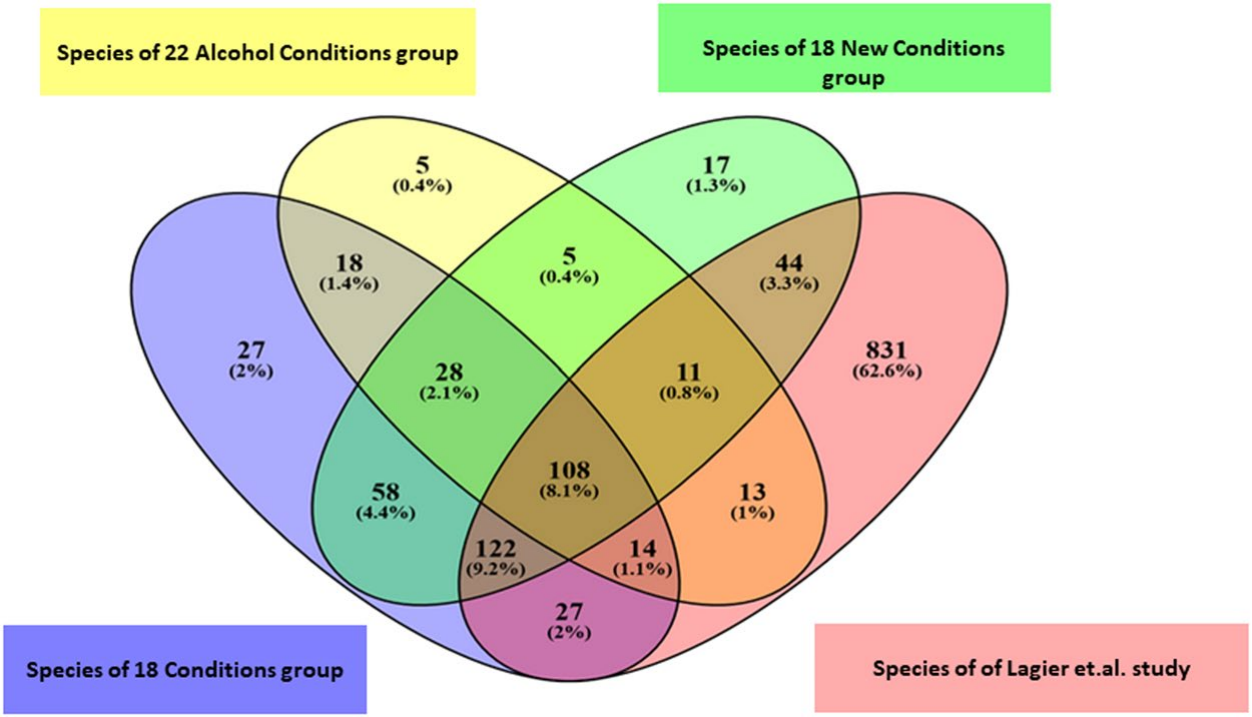
Comparison of the bacterial species in each culture condition group of this study with the list of species isolated in the human gut by culturomics, established by Lagier *et.al*. In yellow the group of 22 alcohol conditions, in green the group of 18 new conditions, in blue the group of 18 previously selected conditions and in pink the species isolated in the study of Lagier *et.al*.

In a second step, we compared our different groups according to species name to the list of 1,170 bacterial species isolated by culturomics in the 2016 study by Lagier *et al*.^6^. We observed that the 18 new conditions group has enabled the addition of 108 species to the list of 1,170 species, of which 17 were specific to this group (Supplementary Fig. 3(b), Fig. 3). Of these 108 species, 58% (n = 63 species) are new species isolated for the first time by culturomics. The group 22 alcohol conditions enabled the addition of 56 species, of which 5 were specific (Supplementary Fig. 3(c), Fig. 3). A large proportion of these 56 species were new taxa (n = 37 species; 66%). The 18 conditions that are already part of the culturomics conditions used in Lagier’s study enabled the addition of 131 species to the list of 1,170 species, of which 27 were specific and 59% (n = 78 species) were culturomics new species (Supplementary Fig. 3(d), Fig. 3). Interestingly, fastidious bacteria such as *Faecalibacterium prausnitzii* and *Akkermansia muciniphila* were isolated in this study, mainly due to the use of new media *(Akkermansia muciniphila* was isolated once in the **HRS Ana 37 °C** condition).

### Comparison between aerobic and anaerobic culture conditions

A total of 40 culture conditions were performed under anaerobic conditions and 18 under aerobic conditions. Overall, the 58 cultures conditions enabled to recover 333 strictly anaerobic species (67%) and 164 (33%) oxygen-tolerant species. Comparing the profitability in terms of species between aerobic and anaerobic culture conditions, we can observe that anaerobic atmosphere condition allowed the isolation of a total of 490 of the 497 species (99%), of which 68% (n = 332 species) were strictly anaerobic and 32% (n = 158 species) oxygen tolerant. While the conditions of culture in aerobic atmosphere allowed the isolation of a total of 89 species, 75% (n = 67 species) were oxygen tolerant and intriguingly 25% (n = 22 species) were strictly anaerobic species. Of the 89 species isolated by aerobic culture conditions, 92% (n = 82 species) were also recovered by anaerobic culture conditions, while only 8% (n = 7 species) were isolated under aerobic conditions only. Of these, only *Bacillus weihenstephanensis* is considered a strict aerobic species. The aerobic conditions, which allowed the isolation of more than 20 oxygen-tolerant bacteria are **Filtration 0,45 µm Ae 37 °C** (n = 32 species), **HR Ae 37 °C** (n = 27 species), **HRS Ae 37 °C** (n = 21 species), **HS Ae 37 °C** (n = 21 species).

### Impact of rumen supplementation

**Figure 4:**
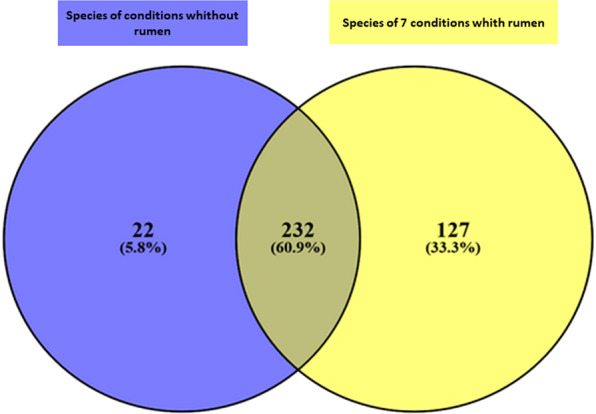
Comparison of bacterial species found in 7 conditions without rumen in blue (Hemoc Ana 37 °C, HS Ae 37 °C, HS Ana 37 °C, HS alcohol Ae 37 °C, HS alcohol Ae 28 °C, HS alcohol Ana 28 °C) against 7 other corresponding conditions with rumen in yellow (HR Ana 37 °C, HRS Ae 37 °C, HRS Ana 37 °C, HRS alcohol Ae 37 °C, HRS alcohol Ana 37 °C, HRS alcohol Ana 37 °C, HRS alcohol Ae 28 °C, HRS alcohol Ana 28 °C).

In order to show the contribution of the rumen supplementation in our study, we selected a group of 14 conditions (7 conditions with rumen and the same 7 conditions without rumen) and compared them with each other. Conditions including rumen fluid enabled to isolate a higher number of bacterial species than the conditions without rumen (n = 359 VS n = 254)). The number of bacterial species specific to conditions with rumen (N = 127; 33%) was significantly higher than those specific to conditions without rumen (Chi-square test, p < 10^−6^), Of these 127 specific species, 61% (n = 77) are strict anaerobes bacteria and 38% (n = 48) are new taxa isolated as a part of culturomics studies (Fig. 4).

## Discussion

The purpose of this study was to optimize and standardize the culture conditions of culturomics. First, the 40 additional culture conditions that were added allowed the isolation of 95 additional species compared to the 18 pre-selected conditions, of which 27% (n = 26) are new culturomics taxa. Among these 95 species, 27 species were not found in the Lagier *et al*. study of which 52% (n = 14) represent new culturomics taxa. This shows the contribution of the new conditions used, particularly in the isolation of new bacterial species. Second, we were able to establish a list of the 15 best culture conditions that each isolate more than 100 bacterial species. When comparing this ranking to the 15 best of the 25 optimized conditions according to their ability to add the greatest number of previously non-isolated species, 12 conditions are found in common (Supplementary Fig. 4). Of the 3 remaining conditions, only *Christensenella* medium Ana37 °C is included in the 25 optimized conditions. The other two conditions (Hemoc Ana 37 °C and HR Ana37 °C), despite their ability to isolate a significant number of species (n = 143 species and n = 141 species), do not allow the cultivation of additional species. Therefore the, *Christensenella* medium Ana37 °C has been added to the 15 best optimized conditions resulting a recommended panel of 16 new conditions. This panel includes 8 of 18 new conditions, 6 of the 18 previously defined conditions and 2 of 22 alcohol conditions (Table 1). These 16 new optimal culturomics conditions allow the recovery of 98% (n = 487 species) of the total species isolated in this study, thereby suppressing 42 unnecessary conditions. Composition of these 16 different culture conditions are detailed in Supplementary Table S2. The blood culture bottle with rumen fluid and sheep blood in anaerobic condition at 37 °C (**HRS Ana 37 °C**) is the condition that allowed to capture the maximal bacterial richness since 306 species (62%) were recovered using this condition only. Regarding the medium composition, we have demonstrated that rumen fluid was required to cultivate a substantial number of bacterial species (Fig. 4). The rumen is a fermentation site found of most ruminants in which there is an accumulation of fermentation products such as acetate or propionate. These latter are included in several formulations of culture media (i.e., YCFA medium). Most of the species found only in conditions supplemented rumen fluid were anaerobes (i.e., 66%). We have previously shown that use of rumen fluid was particularly fertile for discovering new taxa, in particular when the specimen incubated in a blood culture^16^. On the other hand, we were surprised by the fact that 25% of species recovered in aerobic atmosphere are known as strict anaerobes according to the literature. This could be the consequence of a partial tolerance to oxygen that is known to be variable among strains belonging to the same species^17^. Overall, anaerobic culture conditions were superior to aerobic culture conditions as they allow to recover 5 times more bacteria than aerobic culture conditions, which enabled to specifically culture 1% of the bacterial species only. Indeed, as some aerobes were found to grow under anaerobic conditions, the number of aerobic conditions performed should probably be restricted to a strict minimum.

**Table 1:** List of the 16 best culture conditions recommended in this study.

16 Best culture conditions of culturomics	Number of species added and not previously isolated	Number of species isolated
Blood culture bottle with rumen fluid and sheep blood in anaerobic condition at 37 °C (HRS Ana 37 °C)	n = 306	n = 306
R-medium with lamb serum with rumen fluid and sheep blood in anaerobic condition at 37 °C (R-medium-SA- RS Ana37 °C)	n = 64	n = 172
Blood culture bottle with rumen fluid and sheep blood in anaerobic condition at 37 °C after pre-treatment of stool sample with alcohol (HRS Ana 37 °C Alcohol)	n = 29	n = 133
YCFA broth in anaerobic condition at 37 °C (YCFA Ana 37 °C)	n = 21	n = 152
Blood culture bottle with stool filtered at 0,45 µm in anaerobic condition at 37 °C (Filtration 0,45 µm Ana 37 °C)	n = 17	n = 144
Blood culture bottle with 5 ml sheep blood in anaerobic condition at 37 °C (HS Ana 37 °C)	n = 13	n = 166
Blood culture bottle after thermic shock at 80 °C during 20 min in anaerobic condition at 37 °C (TS Ana 37 °C)	n = 8	n = 141
Marine broth in anaerobic condition at 37 °C (Marin Ana37 °C)	n = 5	n = 139
Blood culture bottle with stool filtered at 0,45 µm in aerobic condition at 37 °C (Filtration 0,45 µm Ae 37 °C)	n = 5	n = 35
Blood culture bottle with stool filtered at 5 µm in anaerobic condition at 37 °C (5 µm Ana 37 °C)	n = 4	n = 126
Blood culture bottle with rumen fluid in anaerobic condition at 37 °C after pre-treatment of stool sample with alcohol (HR Ana 37 °C)	n = 4	n = 64
R-medium with rumen fluid and sheep blood in anaerobic condition at 37 °C (R-medium-RS Ana 37 °C)	n = 3	n = 127
CNA agar medium in anaerobic condition at 37 °C (CNA agar Ana 37 °C)	n = 3	n = 50
5% sheep blood broth in anaerobic condition at 37 °C (COS Ana 37 °C)	n = 2	n = 167
Schaedler broth in anaerobic condition at 37 °C (Schaedler Ana 37 °C)	n = 2	n = 123
*Christensenella* broth medium in anaerobic condition at 37 °C (*Christensenella* medium Ana 37 °C)	n = 1	n = 116

Supplementary Table S1. 58 Culture conditions used in this study.

Supplementary Table S2. Detailed composition of the different culture conditions used in this study.

Supplementary Table S3. Donor characteristics.

Supplementary Data 1. List of bacterial species isolated in this study according to the different conditions used.

In the end, we were unable to determine ideal culture conditions for the isolation of certain taxa species because, following our analyses on the subject, we did not find any specific association. The only exception concerned *Faecalibacterium prausnitzii*, which was only recovered using YCFA medium. This reinforces the fact that there is a degree of chance in the isolation of rare bacteria^18^. However, we provide herein a list of all the species isolated in this work including 16S rRNA gene sequences accession numbers and the conditions under which they were isolated necessary (Supplementary Data 1). In any case, this work contributes to standardize the culture-dependant techniques for exploring gut microbiota composition. Other works have developed innovative approaches to reduce the workload of these methods that include a prior analysis of the optimal media to be used^5,13^. If we have test herein different formulas and supplementations, we kept agar as solidifying agent, There are however evidences that gellan gum represents an alternative to agar^19–23^. It is indeed less expensive, is not susceptible to agarases. It would also avoid the production of hydrogen peroxide during the agar autoclaving of the agar in the presence of phosphate, which is toxic for some extremely sensitive to oxygen bacteria. The use of gellan gum as solidifying agent was shown to capture a different bacterial diversity that when agar is used^13^. Outside the medium composition, optimization of the cultures handling or the identification process could be time- and labour saving. Thus, experimental colony picking or massive phylogenetic markers and genome sequencing were experienced with success^5,13^. These standardization processes are essential for optimizing cultural approaches thereby paving the way for bacteriotherapy^24–27^.

## Materials and methods

### Samples

In this work, we have included 8 fresh stools. The specimens were collected from 8 apparently healthy subjects, including several donors included as a part of fecal microbiota transplantation (FMT)^28,29^. The subjects have been living in Marseille for at least one year but may come from different countries. The fresh stool samples were directly inoculated 5 minutes after emission to prevent the loss of certain anaerobic bacteria by preservation. Samples were collected at different times to test the maximum culture condition. The donors have all signed a written consent and the project has been approved by the IHU Mediterranean Infection’s ethics committee under number 2016-011 and informed consent was obtained from all subjects. Individuals did not take antibiotics at least one month preceding the specimen collection. The main information related to the participants are summarized in Supplementary Table S3.

### Culturomics protocol

Culturomics is a high-throughput culture technique consisting in the multiplication of the culture conditions along with a quick bacterial identification using MALDI-TOF MS^1^.

Herein, a total of 58 culture conditions were tested, based on 15 culture media in solid or liquid form with various variations such as atmosphere (aerobic or anaerobic), application of different temperatures (28 °C; 37 °C), pre-treatment with alcohol, addition of rumen fluid and/or sheep blood (Supplementary Table S1).

- The 18 best conditions standard described by culturomics were selected in this study as previously described^6^.
- We have introduced 18 new media and culture conditions, some of which have been used for the selective growth of certain anaerobic, fastidious and slow-growing bacteria such as *Chistensenella* and *Faecalibacterium (i.e. Christensenella* medium, YCFA medium (medium with yeast extract, casitone, fatty acid) specific for the isolation of *Faecalibacterium* species,
- In order to selectively isolate spore-forming bacteria, alcohol conditions were performed. Stool was pre-treated with alcohol to eliminate as much as possible vegetative forms to promote the growth of sporulating bacteria according to the protocol of Afouda *et al*.^30^.

The composition of these 58 culture conditions is detailed in Supplementary Table S2.

After emission, 1 g of each sample is diluted in a 900 µl solution of Dulbecco’s Phosphate-Buffered Saline (DPBS) and then immediately inoculated into a culture flask. Bacterial cultures are monitored for 30 days. Every 3 days, the liquids of blood culture flasks are inoculated with Colombia agar enriched with sheep blood at 5% (bioMérieux, Marcy l’Etoile, France) after series of dilutions. For direct inoculations without pre-enrichment, the samples diluted in phosphate buffered saline (PBS) undergo a series of dilutions before being seeded on the different solid media in accordance with the protocol of Diakite *et al*.^15^.

### Bacterial identification by MALDI-TOF/MS and 16S rRNA gene sequencing

After 24 to 72 hours of incubation, the bacterial colonies obtained are identified by MALDI-TOF according to the protocol described by Seng *et al*.^31^. Bacterial colonies unidentified by MALDI-TOF mass spectrometry were sequenced with 16S rRNA gene, as previously described^32^. Following 16S rRNA gene sequencing, sequences that have a similarity percentage lower than 98.65% are defined as new bacterial species, and those less than 95% as new bacterial genera^33^.These are described according to the taxonogenomic principle described by Fournier *et al*.^34^.

### Phylogenetic tree construction

Phylogenetic tree highlighting of 480 isolated gut bacteria concidering the 16S rRNA was obtained by downloading 16S rRNA gene sequences from Genbank.

Genbank accession numbers of 16S rRNA gene sequences are indicated in parentheses. Sequences were aligned using MUSCLE with default parameters, phylogenetic inference were obtained using the Maximum likelihood method and the fasttree software. The visualization and customization have been realized thanks to the itol platform (https://itol.embl.de/).

All methods were carried out in accordance with relevant guidelines and regulations.

## Supplementary information

Supplementary Information.

Supplementary Information 2.
