# Supplementary material for: Optimization and standardization of the culturomics technique for human microbiome exploration
Source: Sci Rep. 2020 Jun 15;10:9674. doi: 10.1038/s41598-020-66738-8 (PMC7295790; doi:10.1038/s41598-020-66738-8)
Supplement: Supplementary file 1 — Supplementary Information. [file 41598_2020_66738_MOESM1_ESM.pdf]

Optimization and standardization of the culturomics technique for human microbiome exploration.

Ami DIAKITE[1,2], Grégory DUBOURG[1,2], Niokhor DIONE[1,2], Pamela AFOUDA[1,2], Sara BELLALI[1,2], Issa Isaac NGOM[1,2], Camille VALLES[1,2], Mamadou Lamine TALL[1,2], Jean-Christophe LAGIER[1,2] and Didier RAOULT[1,2#]

[1]*Aix Marseille Univ, IRD, AP-HM, MEPHI, Marseille, France*

[2] *IHU Méditerranée Infection, Marseille, France*

#Corresponding author : Didier Raoult[1], Aix Marseille Université, IHU Méditerranée Infection, Marseille, France, E-mail : didier.raoult@gmail.com

Phone number: +33 4 13 73 24 01

Fax number: +33 4 13 73 24 02

Supplementary Fig S1. Classification of culture conditions according to their profitability in number of isolated species

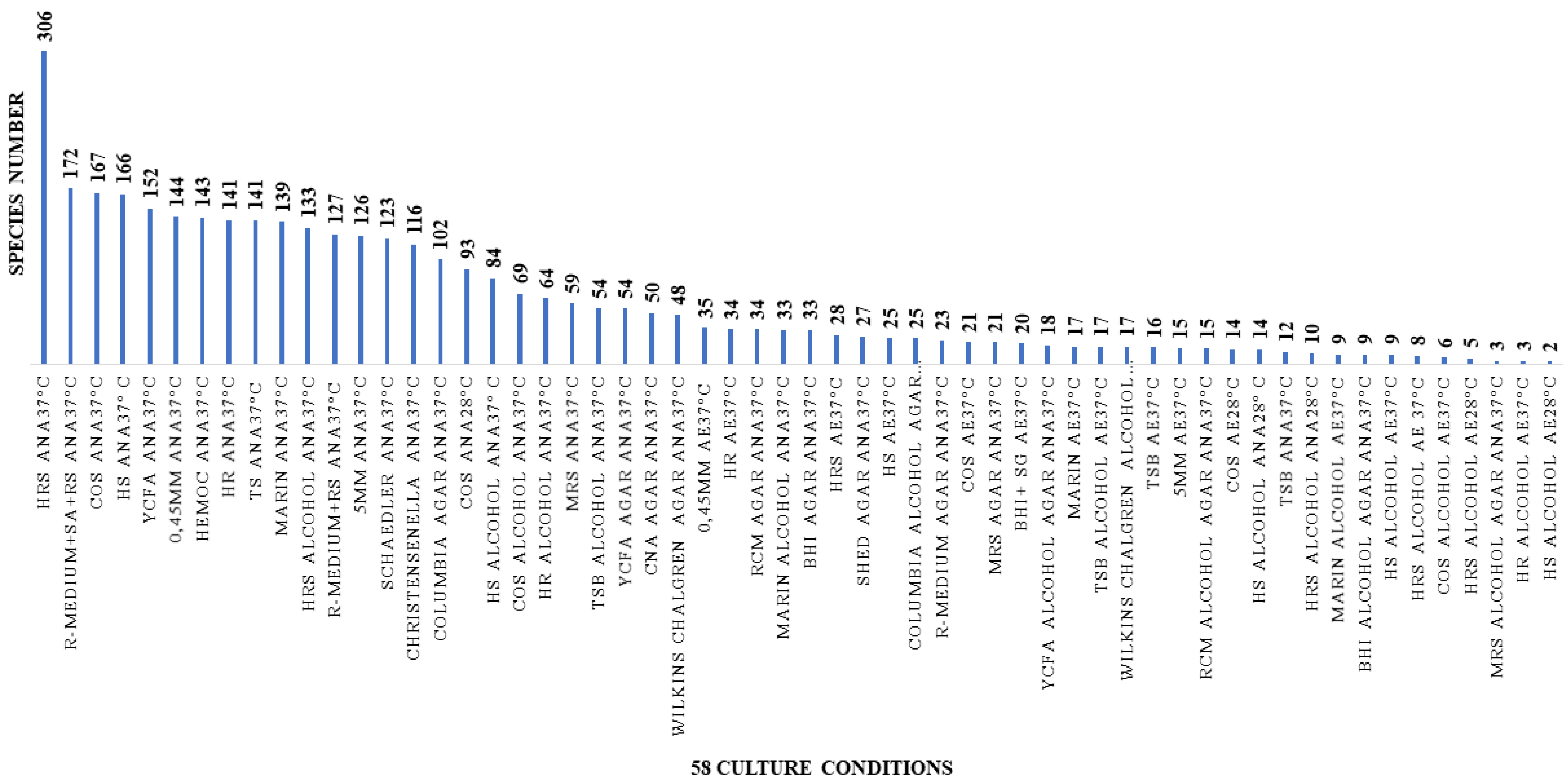

Supplementary Fig S2. Classification of culture conditions according to their ability to add the greatest number of previously non-isolated species by HRS Ana 37°C condition.

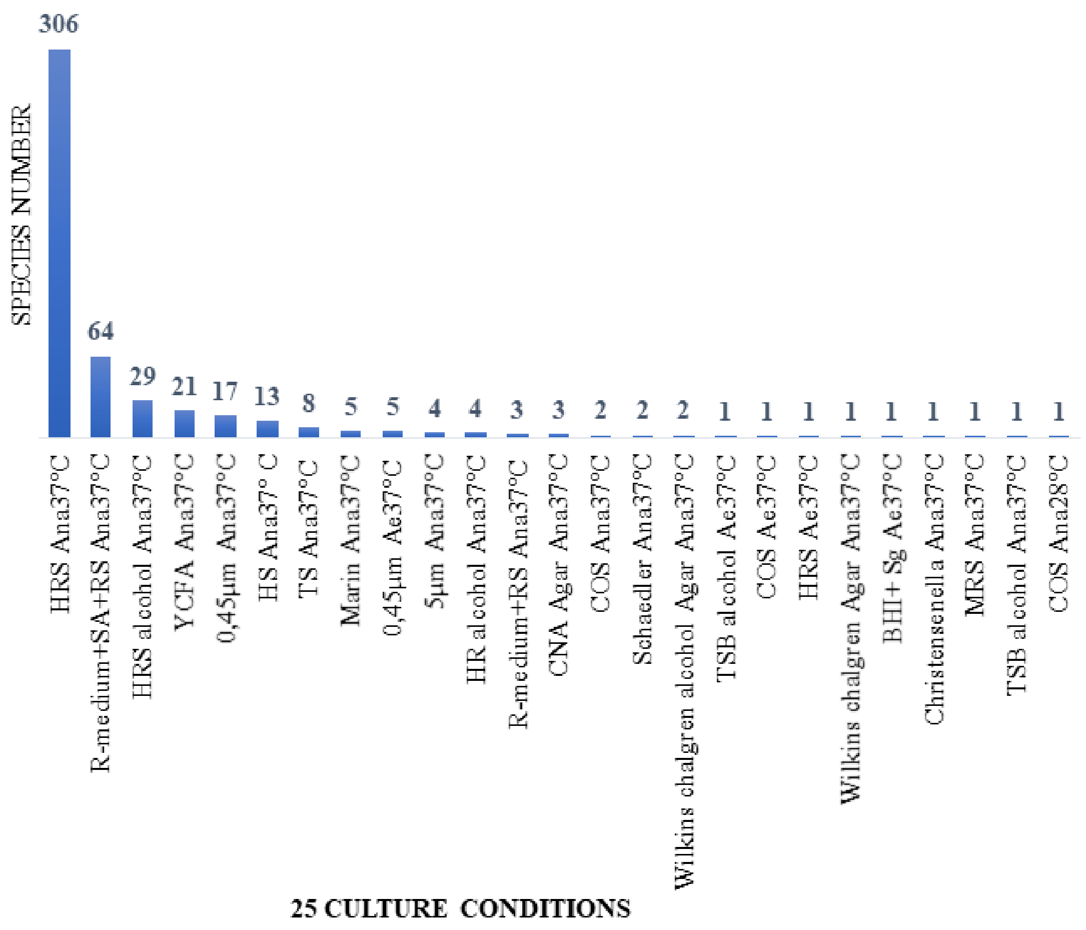

Supplementary Fig S3. Comparison of the bacterial species between the 3 groups of culture conditions: 18 previously selected conditions group, 18 new conditions group and 22 alcohol conditions group **(a)**. Each group was distinctively compared to the list of species isolated from the human gut by culturomics, established by Lagier et al **(b), (c), (d)**.

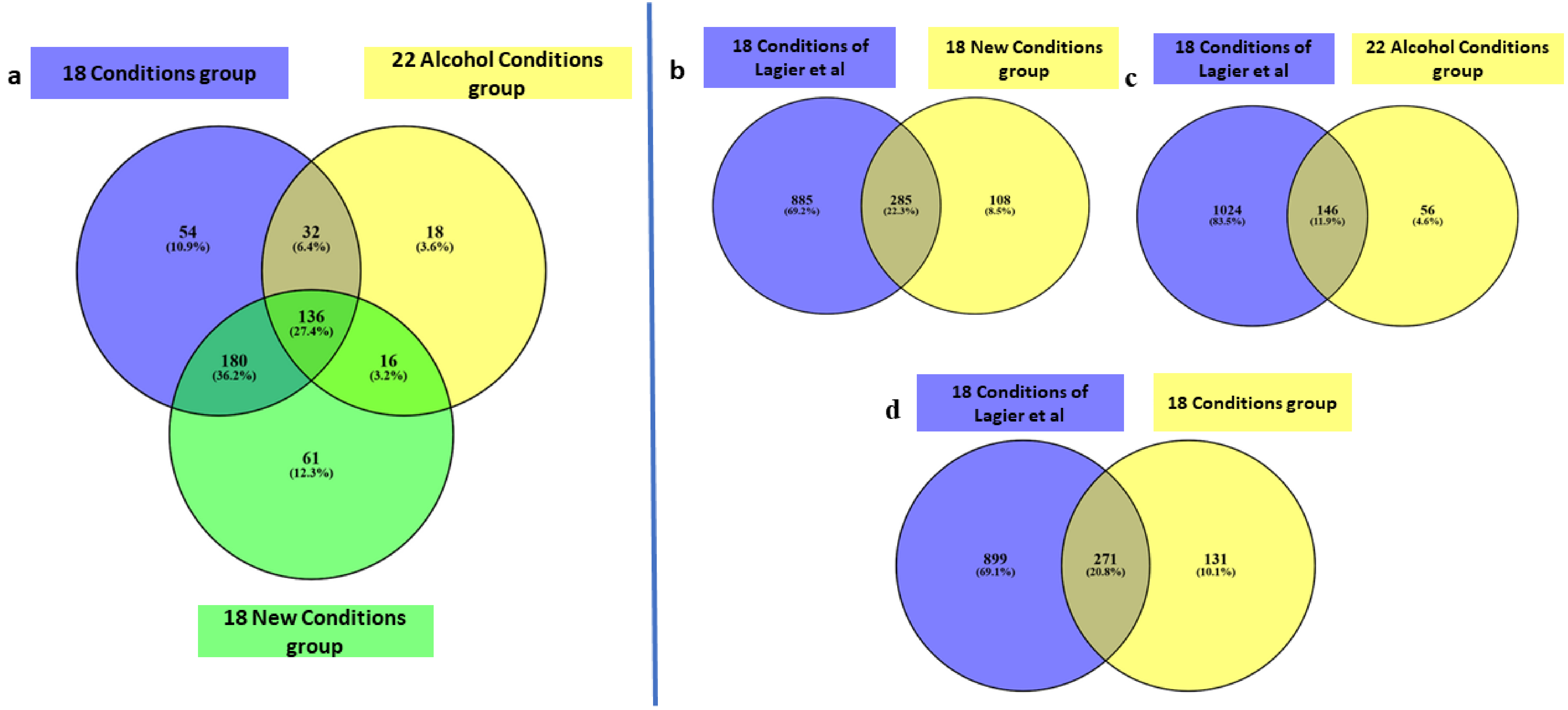

Supplementary Fig. S4: Comparison between the 15 best conditions of 58 totals conditions tested and the 15 best conditions of 25 conditions optimized in this study.

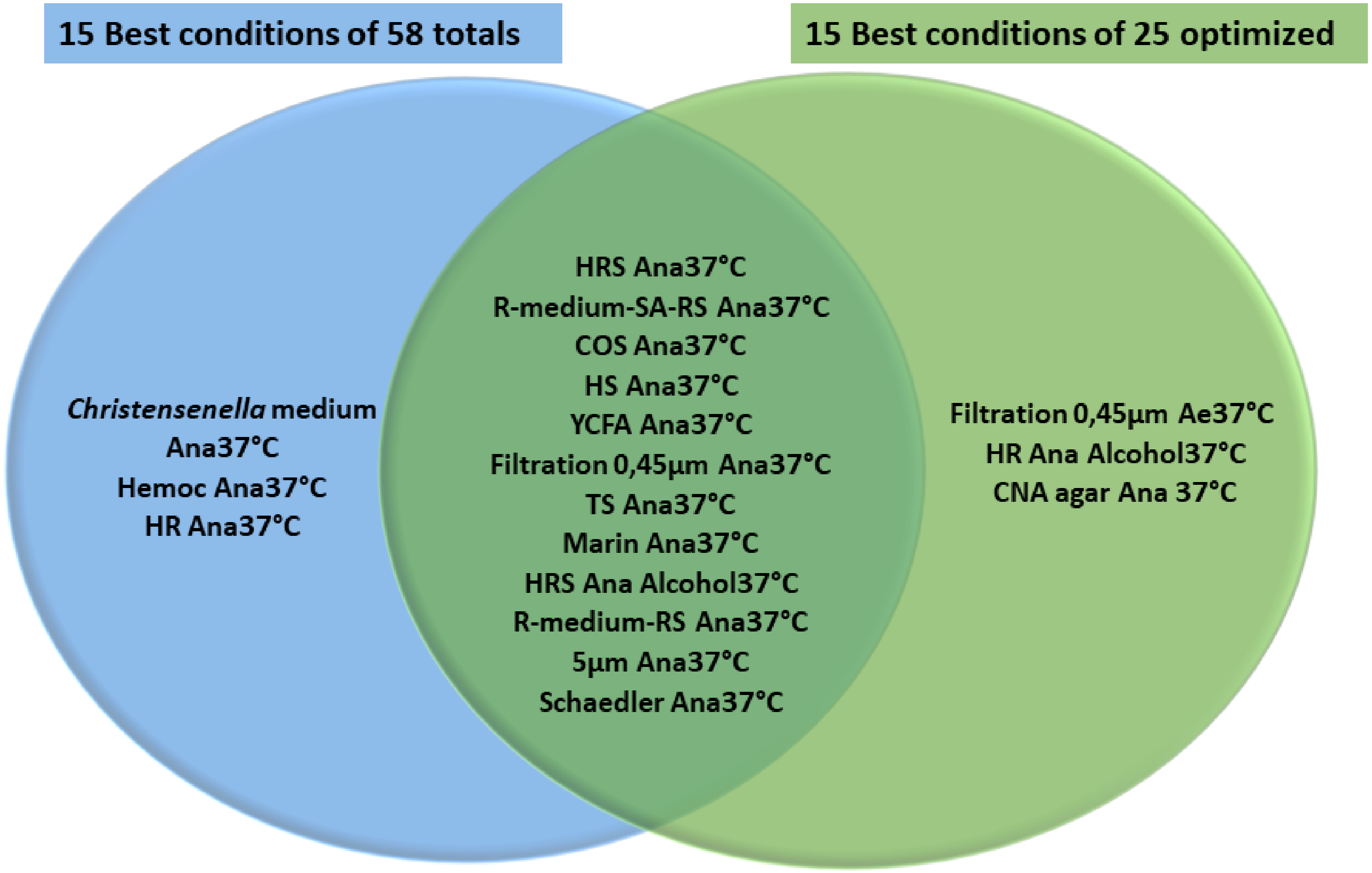

Supplementary table S1: 58 Culture Conditions use in this study.

**18 culturomics standard conditions**
1- Preincubation of stool preparation in **anaerobic blood culture bottle (Hemoc Ana37°C)**
2- Preincubation of stool preparation in **aerobic blood culture bottle** with 5ml of rumen fluid **(HR Ae37°C)**
3- Preincubation of stool preparation in **anaerobic blood culture bottle** with 5ml of rumen fluid **(HR Ana37°C)**
4- Preincubation of stool preparation in **aerobic blood culture bottle** with 5ml of sheep blood **(HS Ae37°C)**
5- Preincubation of stool preparation in **anaerobic blood culture bottle** with 5ml of sheep blood **(HS Ana37° C)**
6- Preincubation of stool preparation in **aerobic blood culture bottle** with 5ml of rumen fluid and 5ml of sheep blood (**HRS Ae37°C**)
7- Preincubation of stool preparation in **anaerobic blood culture bottle** with 5ml of rumen fluid and 5ml of sheep blood (**HRS Ana37°C)**
8- Preincubation of stool preparation filtered at 5µm in **aerobic blood culture bottle (5µm Ae37°C)**
9- Preincubation of stool preparation filtered at 5µm in **anaerobic blood culture bottle (5µm Ana37°C)**
10- Preincubation of stool preparation after thermic shock at 80°C during 20min in **anaerobic blood culture bottle (TS Ana37°C)**
11- Preincubation of stool preparation in **COS liquid medium** with 5% sheep blood in aerobic condition **(Cos Ae37°C)**
12- Preincubation of stool preparation in **COS liquid medium** with 5% sheep blood in anaerobic condition **(Cos Ana37°C)**
13- Preincubation of stool preparation in **COS liquid medium** with 5% sheep blood in aerobic condition **(Cos Ae28°C)**
14- Preincubation of stool preparation in **COS liquid medium** with 5% sheep blood in anaerobic condition **(Cos Ana28°C)**
15- Preincubation of stool preparation in **Marine broth** in aerobic condition (**Marin Ae37°C)**
16- Preincubation of stool preparation in **Marine broth** in anaerobic condition **(Marin Ana37°C)**
17- Preincubation of stool preparation in **Trypticase Soy Broth medium** in aerobic condition **(TSB Ae37°C)**
18- Preincubation of stool preparation in **Brain Heart Infusion broth** with 5% sheep blood in aerobic condition **(BHI+ Sg Ae37°C)**
**New culture media introduced**
1- Preincubation of stool preparation in **YCFA medium** in anaerobic condition **(YCFA Ana37°C)**
2- Preincubation of stool preparation in **Christensenella medium** in anaerobic condition **(Christensenella Ana37°C)**
3- Preincubation of stool preparation in **MRS medium** in anaerobic condition **(MRS Ana37°C)**
4- Preincubation of stool preparation in **Schaedler medium** in anaerobic condition **(Schaedler Ana37°C)**
5- Preincubation of stool preparation in **R-medium and lamb serum** with 5ml of rumen fluid and 5ml of sheep blood in anaerobic condition **(R-medium+SA+RS Ana37°C)**
6- Preincubation of stool preparation in **R-medium** with 5ml of rumen fluid and 5ml of sheep blood in anaerobic condition **(R-medium+RS Ana37°C)**
7- Preincubation of stool preparation filtered at 0,45µm in **aerobic blood culture (0,45µm Ae37°C)**
8- Preincubation of stool preparation filtered at 0,45µm in **anaerobic blood culture bottle (0,45µm Ana37°C)**
9- Preincubation of stool preparation in **Trypticase Soy Broth medium** in anaerobic condition **(TSB Ana37°C)**
10- Direct culture of stool preparation in **CNA Agar medium** in anaerobic condition **(CNA Agar Ana37°C)**
11- Direct culture of stool preparation in **R-medium Agar** in anaerobic condition **(R-medium Agar Ana37°C)**
12- Direct culture of stool preparation in **Schaedler Agar medium** in anaerobic condition **(Shed Agar Ana37°C)**

13- Direct culture of stool preparation in **YCFA Agar medium** in anaerobic condition **(YCFA Agar Ana37°C)**
14- Direct culture of stool preparation in **Reinforced clostridial Agar medium** in anaerobic **(RCM Agar Ana37°C)**
15- Direct culture of stool preparation in **Wilkins chalgren Agar medium** in anaerobic condition **(Wilkins chalgren Agar Ana37°C)**
16- Direct culture of stool preparation in **Brain-heart infusion Agar medium** in anaerobic condition **(BHI Agar Ana37°C)**
17- Direct culture of stool preparation in **Columbia Blood Agar medium** in anaerobic condition **(Columbia Agar Ana37°C)**
18- Direct culture of stool preparation in **MRS Agar medium** in anaerobic condition **(MRS Agar Ana37°C)**
**22 Alcohol condition**
1- Preincubation of stool preparation in **aerobic blood culture bottle** with 5ml of rumen fluid **(HR alcohol Ae37°C)**
2- Preincubation of stool preparation in **anaerobic blood culture bottle** with 5ml of rumen fluid **(HR alcohol Ana37°C)**
3- Preincubation of stool preparation in **aerobic blood culture bottle** with 5ml of sheep blood **(HS alcohol Ae37°C)**
4- Preincubation of stool preparation in **anaerobic blood culture bottle** with 5ml of sheep blood **(HS alcohol Ana37° C)**
5- Preincubation of stool preparation in **aerobic blood culture bottle** with 5ml of sheep blood **(HS alcohol Ae28°C)**
6- Preincubation of stool preparation in **anaerobic blood culture bottle** with 5ml of sheep blood **(HS alcohol Ana28° C)**
7- Preincubation of stool preparation in **aerobic blood culture bottle** with 5ml of rumen fluid and 5ml of sheep blood (**HRS alcohol Ae 37°C**)
8- Preincubation of stool preparation in **anaerobic blood culture bottle** with 5ml of rumen fluid and 5ml of sheep blood (**HRS alcohol Ana37°C)**
9- Preincubation of stool preparation in **aerobic blood culture bottle** with 5ml of rumen fluid and 5ml of sheep blood (**HRS alcohol Ae28°C**)
10- Preincubation of stool preparation in **anaerobic blood culture bottle** with 5ml of rumen fluid and 5ml of sheep blood (**HRS alcohol Ana28°C**)
11- Preincubation of stool preparation in **COS liquid medium** with 5% sheep blood in aerobic condition **(Cos alcohol Ae37°C)**
12- Preincubation of stool preparation in **COS liquid medium** with 5% sheep blood in anaerobic condition **(Cos alcohol Ana37°C)**
13- Preincubation of stool preparation in **Marine broth** in aerobic condition (**Marin alcohol Ae37°C)**
14- Preincubation of stool preparation in **Marine broth** in anaerobic condition **(Marin alcohol Ana37°C)**
15- Preincubation of stool preparation in **Trypticase Soy Broth medium** in aerobic condition **(TSB alcohol Ae37°C)**
16- Preincubation of stool preparation in **Trypticase Soy Broth medium** in anaerobic condition **(TSB alcohol Ana37°C)**
17- Direct culture of stool preparation in **YCFA Agar medium** in anaerobic condition **(YCFA alcohol Agar Ana37°C)**
18- Direct culture of stool preparation in **Reinforced clostridial Agar medium** in anaerobic **(RCM alcohol Agar Ana37°C)**
19- Direct culture of stool preparation in **Wilkins chalgren Agar medium** in anaerobic condition **(Wilkins chalgren alcohol Agar Ana37°C)**
20- Direct culture of stool preparation in **Brain-heart infusion Agar medium** in anaerobic condition **(BHI alcohol Agar Ana37°C)**
21- Direct culture of stool preparation in **Columbia Blood Agar medium** in anaerobic condition **(Columbia alcohol Agar Ana37°C)**
22- Direct culture of stool preparation in **MRS Agar medium** in anaerobic condition **(MRS alcohol Agar Ana37°C)**

Supplementary table S2: Detailed composition of the different culture conditions used in this study.

CULTURE MEDIUM	COMPOSITION
**Blood culture bottle**	Blood culture bottle BACT/ALERT Flacons FAN® Plus<br>(Fastidious Antimicrobial Neutralization) by Biomerieux<br>40 ml of complex medium supplemented with absorbent polymeric beads<br>Aerobic Ref. 410851<br>Anaerobic Ref. 410852
**COS liquid medium 5% sheep blood**	Yeast Extract. .... 5 g<br>Peptone. .... 5 g<br>Brain Heart Infusion .... 10 g<br>NH_4CL. .... 1 g<br>K_2HPO_4 .... 0.3 g<br>KCL. .... 0.3 g<br>Ascorbic Acid: .... 1 g<br>Sheep Blood: .... 5%<br>Distilled Water.......... 1000 ml
**Marine broth**	Peptone. .... 5 g<br>Yeast Extract. .... 1 g<br>Ferric Citrate.......... 0.1 g<br>Sodium Chloride.......... 19.45 g<br>Magnesium Chloride .... 8.8 g<br>Sodium Sulfate. .... 3.24 g<br>Calcium Chloride.......... 1.8 g<br>Potassium Chloride.......... 0.55 g<br>Sodium Bicarbonate. .... 0.16 g<br>Potassium Bromide.......... 0.08 g<br>Strontium Chloride .... 34 mg<br>Boric Acid. .... 22 mg<br>Sodium Silicate.......... 4 mg<br>Sodium Fluoride .... 2.4 mg<br>Ammonium Nitrate.......... 1.6 mg<br>Disodium Phosphate. .... 8 mg<br>Distilled Water.......... 1000 ml

**Trypticase Soy Broth**	Tryptone (Pancreatic Digest of Casein)…...................17 g<br>Soytone (Peptic Digest of Soybean)…...................3 g<br>Glucose (Dextrose)…...................2.5 g<br>Sodium Chloride (NaCl)…...................5 g<br>Dipotassium Phosphate (K_2HPO_4)…...................2.5 g<br>Distilled Water...................1000 ml<br>pH 7.3 [illegible] 0.2
**Brain Heart Infusion broth**	Calf Brains, infusion from....................200 g<br>Beef Hearts, infusion from....................250 g<br>Proteose Peptone....................10 g<br>Dextrose...................2 g<br>NaCl...................5 g<br>Na2HPO4....................2.5 g<br>Distilled Water...................1000 ml<br>pH 7.4 +/- 0.2<br>For solid media add agar to the composition
**YCFA medium (modified)**	Casitone...................10 g<br>Yeast Extract…...................2.50 g<br>Glucose...................5 g<br>MgSO4 x 7 H2O…...................45 mg<br>CaCl2 x 2 H2O…...................90 mg<br>K2HPO4…...................0.45 g<br>KH2PO4…...................0.45 g<br>NaCl…...................0.9 g<br>Resazurin…...................1 mg<br>NaHCO3…...................4 g<br>L-Cysteine-HCl...................1 g<br>Hemin…...................10 mg<br>Volatile fatty acids:<br>Acetic Acid…...................1.90 ml<br>Propionic Acid…...................0.70 ml<br>iso-Butyric Acid…...................90 µl<br>n-Valeric Acid…...................100 µl<br>iso-Valeric Acid…...................100 µl<br>Vitamin solution:

	Biotin… ...2 mg<br>Folic Acid… ...2 mg<br>Pyridoxine-HCl...10 mg<br>Thiamine-HCl x 2 H2O ...5 mg<br>Riboflavin… ...5 mg<br>Nicotinic Acid… ... 5 mg<br>D-Ca-pantothenate…...5 mg<br>Vitamin B12… ...0.10 mg<br>p-Aminobenzoic Acid…... 5 mg<br>Lipoic Acid… ...5 mg<br>Distilled Water...1000 ml<br>pH to 6.7 -6.8<br>For solid media add agar to the composition
***Christensenella* medium**	Meat filtrate ... 600 ml<br>Casitone ...30 g/l<br>Yeast extract… ...5 g/l<br>Na-resazurin solution (0.1% w/v)...0,5 ml/l<br>L-Cysteine-HCl x H2O...0,5 g/l<br>Sodium carbonate(Na2CO3) ... 1,5 g/l<br>K2HPO4… ...5 g/l<br>Meat filtrate… ... 400 ml<br>D-Glucose...4 g/l<br>Cellobiose ...1 g/l<br>Maltose ...1 g/l<br>Starch (soluble)... 1 g/l
**MRS-Lactobacillus medium**	Proteose Peptone… ... 10 g<br>Beef Extract....10 g<br>Yeast Extract. ... 5 g<br>Dextrose...20 g<br>Sorbitan Monooleate. ...1 g<br>Ammonium Citrate.... 2 g<br>Sodium Acetate ...5 g<br>MnSO4 xH2O...0.05 g<br>Na2HPO4. ...2 g<br>Distilled Water...1000 ml<br>pH 6.5 +/-0.2

	For solid media add agar to the composition
**Schaedler medium**	Enzymatic Digest of Casein. .... 5.6 g<br>Enzymatic Digest of Soybean Meal. .... 1 g<br>Enzymatic Digest of Animal Tissue. .... 5 g<br>Yeast Extract. .... 5 g<br>Sodium Chloride .... 1.7 g<br>Potassium Phosphate. .... 0.82 g<br>Dextrose .... 5.82 g<br>Tris (hydroxymethyl) Aminomethane .... 3 g<br>Hemin. .... 0.01 g<br>L-Cystine .... 0.4 g<br>Distilled Water .... 1000 ml<br>pH: 7.6 ± 0.2 at 25°C<br>For solid media add agar to the composition
**R-medium**	Casein Hydrolysate .... 15 g<br>Proteose Peptone… .... 15 g<br>Yeast Extract… .... 10 g<br>α-cétoglutarate… .... 2 g<br>NaCl… .... 5 g<br>Na2S .... 0.5 g<br>Haemin… .... 0.1 g<br>K2HPO4… .... 0,83g<br>Glucose .... 10 g<br>L-Cystéine .... 0,5 g<br>Uric acid… .... 0.4 g<br>Ascorbic acid .... 1 g<br>Glutathion… .... 0.1 g<br>Distilled Water .... 1000 ml<br>pH : 7.5<br>For solid media add agar to the composition
	Casein Hydrolysate .... 15g<br>Proteose Peptone… .... 15g<br>Yeast Extract… .... 10g<br>α-cétoglutarate… .... 2g<br>NaCl… 5g

**R-medium and lamb serum**	Na2S .... 0.5 g<br>Haemin… .... 0.1 g<br>K2HPO4… .... 0,83g<br>Glucose .... 10g<br>L-Cystéine .... 0,5g<br>Uric acid … .... 0.4g<br>Ascorbic acid .... 1g<br>Glutathion… .... 0.1 g<br>Lamb Serum… .... 150 ml<br>Distilled Water .... 1000 ml<br>pH : 7.5
**CNA solid medium**	Pancreatic Digest of Casein… .... 12 g<br>Peptic Digest of Animal Tissue .... 5 g<br>Yeast Extract… .... 3 g<br>Beef Extract… .... 3 g<br>Corn Starch… .... 1 g<br>Sodium Chloride .... 5 g<br>Colistin… .... 10 mg<br>Nalidixic Acid… .... 10 mg<br>Sheep Blood, defibrinated… .... 5%<br>Agar… .... 13.5 g<br>Distilled Water .... 1000 ml
**Reinforced clostridial solid medium**	Peptone .... 10 g<br>Beef Extract .... 10 g<br>Yeast Extract .... 3 g<br>Dextrose .... 5 g<br>Sodium Chloride .... 5 g<br>Soluble Starch .... 1 g<br>Cysteine HCl .... 0.5 g<br>Sodium Acetate .... 3 g<br>Agar .... 13.5 g<br>Distilled Water .... 1000 ml<br>pH : 6.8±0.2
	Tryptone. .... 10 g<br>Gelatin Peptone. .... 10 g

**Wilkins chalgren solid medium**	Yeast Extract. .... 5 g<br>Glucose .... 1 g<br>Sodium Chloride .... 5 g<br>L-Arginine .... 1 g<br>Sodium Pyruvate. .... 1 g<br>Menadione. .... 0.0005 g<br>Haemin. .... 0.005 g<br>Agar .... 10 g<br>Distilled Water .... 1000 ml<br>pH 7.1 +/-0.2.
**Columbia Agar medium**	Pancreatic Digest of Casein .... 12.0 g<br>Peptic Digest of Animal Tissue .... 5.0 g<br>Yeast Extract .... 3.0 g<br>Beef Extract .... 3.0 g<br>Corn Starch .... 1.0 g<br>Sodium Chloride .... 5.0 g<br>Agar .... 13.5 g<br>Sheep Blood, Defibrinated .... 5 %<br>pH 7.3 ± 0.2

- Agar was autoclaved separately before being added to the broths.

- Sheep rumen is transported from the slaughterhouse to our laboratory. The digestive contents of sheep stomachs are filtered successively through a funnel covered with a laboratory charlotte. The solid contents are discarded. The suspension will be centrifuged at 10,000 rpm for 90 minutes, the supernatant will be collected for 3 successive filtrations at 0.8µm, 0.45µm, 0.2µm. We thus obtain our rumen juice which can be stored at 4°C.

Supplementary table S3: Donor characteristics.

Donors	Size (m)	Weight (Kg)	Body mass index	Age (years)	Sex	Origin
**Megagut 1**	1,65	60	22	28	Female	French
**Megagut 2**	1,80	72	22,2	32	Male	Senegalese
**Megagut 3**	1,84	76	22,4	32	Male	Senegalese
**Megagut 4**	1,75	55	18	27	Female	Beninese
**Megagut 5**	1,79	95	29,6	32	Male	Cameroon
**Megagut 6**	1,7	69	23,6	30	Male	French
**Megagut 7**	1.65	70	25,7	27	Female	Algerian
**Megagut 8**	1.78	82	25,9	27	Male	Algerian
